# Mobile Apps for Drug–Drug Interaction Checks in Chinese App Stores: Systematic Review and Content Analysis

**DOI:** 10.2196/26262

**Published:** 2021-06-15

**Authors:** Chunying Shen, Bin Jiang, Qilian Yang, Chengnan Wang, Kevin Z Lu, Meng Gu, Jing Yuan

**Affiliations:** 1 Department of Pharmacy Minhang Hospital Fudan University Shanghai China; 2 Department of Pharmacy Administration & Clinical Pharmacy School of Pharmaceutical Sciences Peking University Beijing China; 3 Department of Clinical Pharmacy & Pharmacy Practices School of Pharmacy Fudan University Shanghai China; 4 University of South Carolina College of Pharmacy Columbia, SC United States

**Keywords:** drug interaction, MARS, app, drug safety, drugs, mHealth

## Abstract

**Background:**

As a computerized drug–drug interaction (DDI) alert system has not been widely implemented in China, health care providers are relying on mobile health (mHealth) apps as references for checking drug information, including DDIs.

**Objective:**

The main objective of this study was to evaluate the quality and content of mHealth apps supporting DDI checking in Chinese app stores.

**Methods:**

A systematic review was carried out in November 2020 to identify mHealth apps providing DDI checking in both Chinese iOS and Android platforms. We extracted the apps’ general information (including the developer, operating system, costs, release date, size, number of downloads, and average rating), scientific or clinical basis, and accountability, based on a multidimensional framework for evaluation of apps. The quality of mHealth apps was evaluated by using the Mobile App Rating Scale (MARS). Descriptive statistics, including numbers and percentages, were calculated to describe the characteristics of the apps. For each app selected for evaluation, the section-specific MARS scores were calculated by taking the arithmetic mean, while the overall MARS score was described as the arithmetic mean of the section scores. In addition, the Cohen kappa (κ) statistic was used to evaluate the interrater agreement.

**Results:**

A total of 7 apps met the selection criteria, and only 3 included citations. The average rating score for Android apps was 3.5, with a minimum of 1.0 and a maximum of 4.9, while the average rating score for iOS apps was 4.7, with a minimum of 4.2 and a maximum of 4.9. The mean MARS score was 3.69 out of 5 (95% CI 3.34-4.04), with the lowest score of 1.96 for Medication Guidelines and the highest score of 4.27 for MCDEX mobile. The greatest variation was observed in the information section, which ranged from 1.41 to 4.60. The functionality section showed the highest mean score of 4.05 (95% CI 3.71-4.40), whereas the engagement section resulted in the lowest average score of 3.16 (95% CI 2.81-3.51). For the information quality section, which was the focus of this analysis, the average score was 3.42, with the MCDEX mobile app having the highest score of 4.6 and the Medication Guidelines app having the lowest score of 1.9. For the overall MARS score, the Cohen interrater κ was 0.354 (95% CI 0.236-0.473), the Fleiss κ was 0.353 (95% CI, 0.234-0.472), and the Krippendorff α was 0.356 (95% CI 0.237-0.475).

**Conclusions:**

This study systematically reviewed the mHealth apps in China with a DDI check feature. The majority of investigated apps demonstrated high quality with accurate and comprehensive information on DDIs. However, a few of the apps that had a massive number of downloads in the Chinese market provided incorrect information. Given these apps might be used by health care providers for checking potential DDIs, this creates a substantial threat to patient safety.

## Introduction

Medications are generally safe when used appropriately, but there are risks associated with medication use. Adverse drug event (ADEs), defined as injuries caused by the use of a drug [1], have emerged as a serious public health problem [2]. Every year in the United States, approximately 1 million emergency department visits and 125,000 hospital stays are related to ADEs [3]. Even though detailed data in China are largely lacking, the adverse outcomes caused by drug–drug interactions (DDIs) in China have been estimated to be more serious compared to those in other developed countries, such as the United States. An ADE can be related to a medication error, a DDI, or an adverse drug reaction. As the major contributor to ADEs [1,4], DDIs occur when one drug interferes with another [5], resulting in an altered clinical effect of one or both drugs. DDIs are associated with harmful outcomes, including hospitalizations, emergency department visits, and even death [6,7].

DDIs are avoidable, however, and preventing DDI remains a patient safety challenge in many countries including China. It has been widely reported that physicians and pharmacists, who are in the front line of detecting DDIs, cannot recognize clinically important DDIs [8]. With 20,000 drugs being approved every year, information on more than 100,000 potential DDIs is available in the major medication knowledge bases, such as Micromedex and Lexi-Interact. It is impossible for health care professionals to remember and identify all DDIs. In recent years, medication-related clinical decision support systems have been developed and implemented in some countries to detect and avoid potential DDIs. However, such a system is not common in clinical practice in China. Furthermore, medication knowledge bases often request fees and cannot be easily accessed by Chinese health care professionals, especially those practicing in rural areas.

With the advance of smartphones and mobile apps, using mobile health (mHealth) apps with a DDI checking function seems promising, especially with consideration to the convenience of searching for drug information. However, mHealth apps supporting DDI checks are not subject to the National Medical Products Administration (NMPA) regulations, posing a substantial threat to patient safety. A Canadian study recently reported the results of an assessment evaluating mHealth apps supporting DDI checks and found a lack of high-quality apps [9]. Additionally, the comprehensiveness and quality of app contents underpinning the best available evidence are rarely assessed, especially in non–English-speaking countries [10].

To our best knowledge, there is no published study evaluating the DDI-related mHealth apps available in Chinese app stores. As using incorrect drug information can have serious consequences, the aim of this study was to systematically evaluate DDI-related mHealth apps in Chinese app stores.

## Methods

This review followed the PRISMA (Preferred Reporting Items for Systematic Reviews and Meta-Analyses) systematic review protocol [11,12].

### Search Strategy

We used the “crawling” method to interact directly with app stores’ mHealth repository to avoid any personalized search results that might have been determined by a previous search query. Compared to traditional methods using the search query, creating a health-related app repository allowed us to perform a more thorough and reliable search [10,13]. The crawling method has been applied in searching for weight management [14] and nutrition-related apps [15]. First, we created an app repository by crawling all apps from the health and fitness category in the Chinese iTunes app store webpage (Apple Inc) [16] and the health category in the Chinese Android app store webpage (Google) [17] on November15, 2020. We then extracted the detailed information for the apps that were crawled from the app stores, including the app name, description, user rating, and the number of downloads [16]. Two raters, CW and GM, screened the description of apps independently to select those that supported DDI checks.

To avoid potential omissions, we also carried out an extensive keyword search in both iOS and Android app stores. The search terms included “drug interaction*,” “pill interaction*,” “medication interaction*,” and “DDI*,” following previous app review studies [9]. The inclusion criteria were the following: updated after 2018 and health care professionals as the targeted users. The exclusion criteria were the following: without any DDI information, duplicate apps, and not available in Mandarin Chinese.

CW and MG independently searched in an iPhone or Android phone and selected the apps for inclusion according to the selection criteria. In situations where there was a discrepancy, a third senior rater (CYS) reviewed the app description, and a consensus was made after a thorough discussion. All 3 raters are pharmacists who are currently practicing in the hospital.

### Data Extraction

We first extracted data on the general information about the apps, including the developer, operating system (iOS, Android, or both), costs (free or paid), release date, size (in megabytes), number of downloads, and average rating in the app stores. For mHealth apps supporting a DDI check, the information quality and content accountability are critical for patient safety. Hence, we also extracted specific information related to the scientific or clinical basis and accountability [10], according to the multidimensional framework for the evaluation of apps. 

The scientific or clinical basis refers to the scientific quality of the content and was evaluated by the following metrics: accordance with existing evidence (yes or no), presence of citations (yes or no), clinician involvement (yes or no), affiliation with credible organization (yes or no), and expert assessment (yes or no) [10].

Accountability relates to the credibility of the app developer and was assessed by the presence of the following information: regulatory approval (yes or no), copyright information (yes or no), date of the last update, contact information, and disclaimer [10].

### Quality Assessment

To assess the different factors related to app quality, we used the Mobile App Rating Scale (MARS), a multidimensional instrument for systematically evaluating the quality of mHealth apps [18]. The MARS is a validated tool for evaluating the quality of mHealth apps [18] and has been used in a wide range of fields, such as cardiovascular disease [19], rheumatology [20], mental health, diabetes [21], pediatrics [22], and weight management [14]. MARS is a 23-item expert-based evaluation tool, consisting of multiple dimensions to evaluate different aspects of apps, including end-user engagement, functionality, aesthetics, and information quality [18]. Each question uses a 5-point Likert type scale with a range from 1 to 5 (1 indicates inadequate and 5 indicates excellent; Multimedia Appendix 1). MARS has demonstrated high internal consistency and strong interrater reliability [18]. Procedures performed in previous research informed our method for evaluating the accuracy and comprehensiveness of DDI information [9,23-27]. We used a list of 35 DDI pairs, including 28 true-positive and 7 false-positive examples as the objective example in MARS (Multimedia Appendix 2). Based on clinical pharmacists’ input, we selected these drug combinations because they have significant clinical impact and are commonly used in the existing questionnaires testing clinicians’ knowledge of DDIs [9,23-27]. Before rating the apps, all raters read the MARS protocol and viewed the training video, following the MARS developer’s recommendations [18]. In addition, all raters reached a consensus on the evaluation of the first app.

### Statistical Analysis

Descriptive statistics, including numbers and percentages, were calculated to describe the characteristics of the apps. For each app selected for evaluation, the section-specific MARS scores were calculated by taking the arithmetic mean, while the overall MARS score were calculated as the arithmetic mean of the section scores [18]. In addition, overall scores and section-specific MARS scores were also described by their mean, median, and IQR. The interrater agreement was examined by Cohen kappa coefficient (κ) [28] according to following scoring scheme informed by Cohen and more recent analysis [29]: κ ≤0 indicated no agreement; 0.01-0.20 indicated poor agreement, 0.21-0.40 indicated fair agreement, 0.41-0.60 indicated moderate agreement, 0.61-0.80 indicated substantial agreement, and 0.81-1.00 indicated almost perfect agreement. Spearman correlation was calculated for the relationships among 4 sections of the MARS score, price, and number of downloads. A significance level of *P*<.05 was used in this study. All analyses were performed using SAS 9.4 (SAS Institute).

## Results

### Systematic Search Results

**Figure 1:**
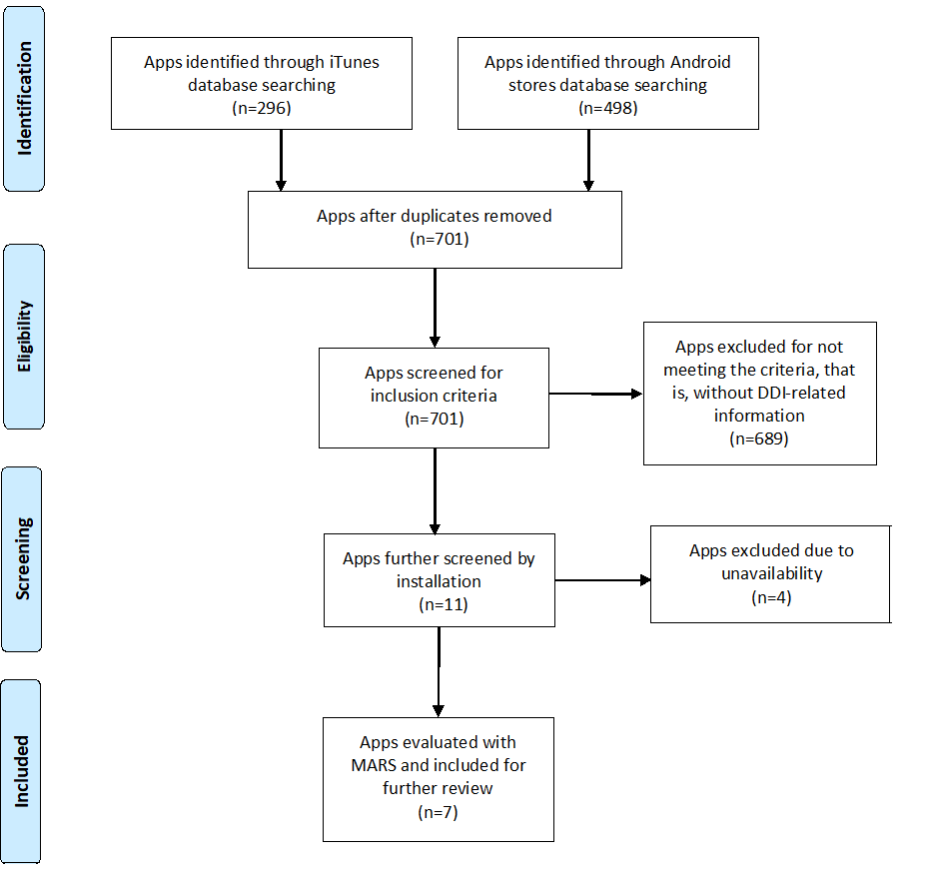
Flowchart of the mobile health app selection. DDI: Drug–Drug Interaction; MARS: Mobile App Rating Scale.

A total of 296 and 498 apps were identified in the iOS and Android App stores, respectively (Figure 1). After duplicates were removed, 701 apps remained. Of these, 689 apps were removed because they did not contain DDI information, and 4 were removed because they could not be downloaded, leaving a total of 7 apps for this evaluation.

### Characteristics of mHealth Apps

Table 1 summarizes the general information of the apps. Out of the 7 apps included in the analysis, 6 (86%) were available free of charge. The MCDEX mobile app provides a 7-day free trial at a price of ¥89 yuan renminbi (US $13.97) per month after trial. Two apps, Medication Assistant by DXY and Medication Assistant of People's Health, offer a free version with limited access to DDI information, with a VIP subscription providing more drug information costing up to ¥30 yuan renminbi (or around US $4.71) per month. Six apps were available on both the iOS and Android platforms, and 1 app was only available on the Android platform. The average size of apps was 108.6 MB, ranging from 2.38 MB to 334.96 MB. The release dates for the Android apps were from January 29, 2012, to June 9, 2017.

Of the 6 apps with a download count available, 3 (50%) had been downloaded more than 1 million times. The average rating score for android apps was 3.5, with a minimum of 1.0 and a maximum of 4.9, while the average rating score for iOS apps was 4.7, with a minimum of 4.2 and a maximum of 4.9.

**Figure 2:**
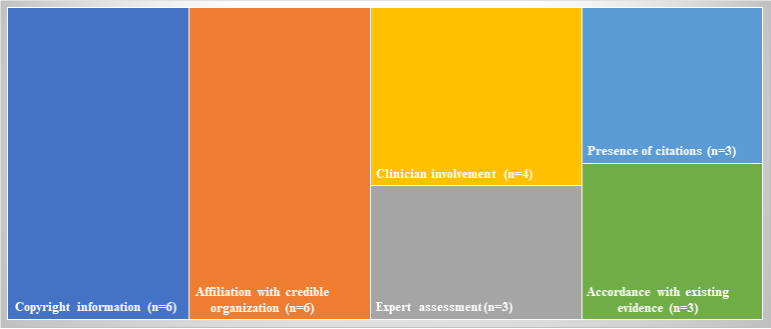
Information quality and accountability of the mobile health apps.

As shown in Figure 2, 3 (43%) of the 7 apps indicated sources of information (ie, citations) and provided information which was in accordance with existing evidence. Of the 7 apps, 4 (57%) indicated the involvement of clinicians in the app development, 3 (43%) had been assessed by experts in the related field, and 6 (86%) were affiliated with credible organizations. None of the apps received regulatory approval, but the MCDEX was developed under the Committee of Experts on Rational Drug Use of the National Health Commission of China, and Medication Assistant of People's Health was affiliated with the People’s Medical Publishing House Co, Ltd, which is the leading professional medical publishing company in China. The date of the latest app update ranged from March 3, 2020, to November 15, 2020. Two apps, Medication Reference and Yi Mai Tong, were developed by the same developer. The detailed information of the apps is presented in Multimedia Appendix 3.

**Table 1:** General information on the mobile health apps included in the review^a^.

App name in English	Target market	Platform	Size (MB)	Cost per month, RMB^b^ (USD)	Release date	Downloads, n^c^	Mean user rating	
							Android	iOS
MCDEX mobile	Health care professionals	iOS & Android	13.06	89 (13.97)	3/5/2015	11,193	3.4	4.2
Medication Assistant by DXY	Health care professionals	iOS & Android	169.16	30 (4.71)	11/17/2012	55,585,879	4.5	4.8
Medication Reference	Doctors, pharmacists, and other HCPs^d^	iOS & Android	334.96	free	7/10/2012	—^e^	4.9	4.5
Medication Assistant of People's Health	Doctors, pharmacists, nurses, and other HCPs	iOS & Android	86.52	12 (1.88)	6/9/2017	227,021	2.0	4.9
Medication Guidelines	Health care professionals	Android	2.38	free	—	750,760	1.0	—
DXY	Health care professionals	iOS & Android	262.67	free	1/29/2012	66,298,525	4.7	4.9
Yi Mai Tong	Health care professionals	iOS & Android	73.67	free	9/26/2013	8,900,870	4.1	4.8

^a^Apps in Chinese app stores were searched on November 15, 2020.

^b^RMB: yuan renminbi.

^c^Number of downloads was not available for the iOS platform.

^d^HCP: health care provider.

^e^Data not available.

### Quality Assessment

**Figure 3:**
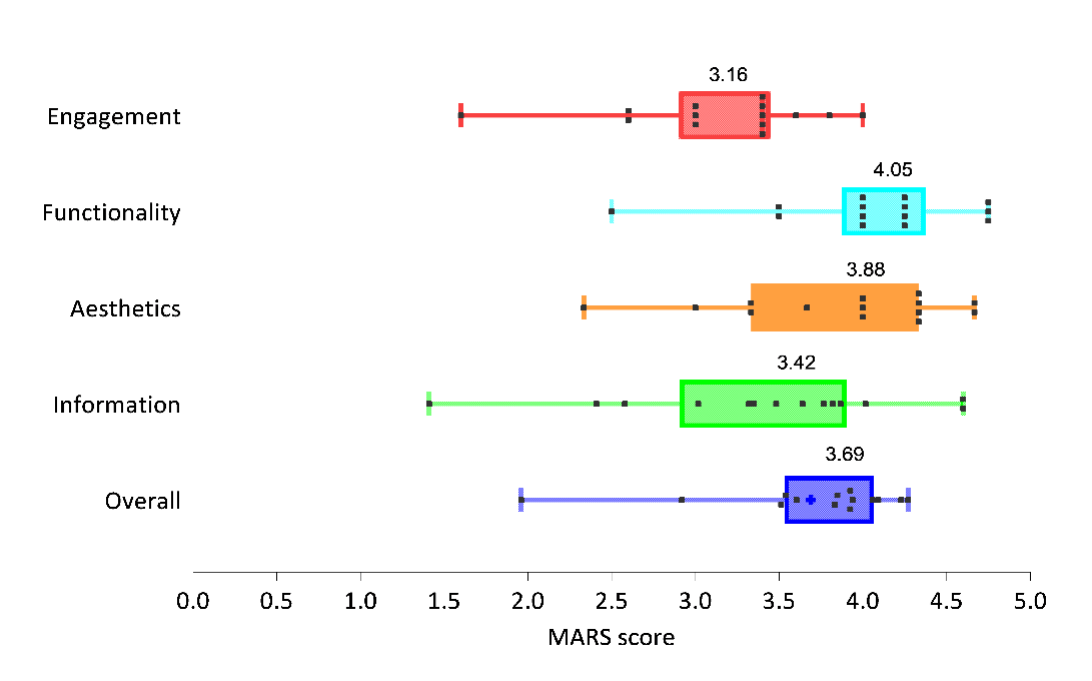
MARS scores by section. The box plot shows the mean, IQR, minimum, and maximum scores. The left and right edge of the boxes represent the first and third quartiles, the line within the boxes represents the mean, and the left and right whiskers represents the minimum and maximum scores. The scatter plot shows the distribution of MARS scores evaluated by 2 raters. MARS: Mobile App Rating Scale.

Overall, the mean MARS score was 3.69 (95% CI 3.34-4.04; Figure 3), with a lowest score of 1.96 for Medication Guidelines and a highest score of 4.27 for MCDEX mobile (Multimedia Appendix 4). A substantial variation in the MARS scores was observed among the 4 sections, with the greatest variation being observed in the information section, ranging from 1.41 to 4.60. The functionality section showed the highest mean score of 4.05 (95% CI 3.71-4.40), whereas the engagement section had the lowest average score of 3.16 (95% CI 2.81-3.51).

For the information quality section, which was the focus of this analysis, the average score was 3.42; the MCDEX mobile app had the highest mean score of 4.6, while the Medication Guidelines app had the lowest average score of 1.9. Of note, in the evaluation of information accuracy, the average score was 2.2 out of 5, or only 44% of the DDI pairs were described correctly. Only 1 of the 7 apps (14%), MCDEX, identified all 35 DDI pairs correctly, while 4 (57%) failed to describe half of the DDI pairs. For those DDI pairs with interactions, the average score was 2.35 out of 5, while the average score was 1.70 out of 5 for those drug pairs without DDIs, indicating these apps had a relatively higher false-positive rate. In the evaluation of the comprehensiveness of information, the average score was 2.9. Out of the 7 apps, 6 (86%) provided incomplete DDI information, while 4 apps covered less than half of the DDIs. The detailed results are presented in Multimedia Appendix 4.

For the overall MARS score, the κ coefficient was 0.354 (95% CI 0.236-0.473), the Fleiss κ was 0.353 (95% CI 0.234-0.472), and the Krippendorff α was 0.356 (95% CI 0.237-0.475). Based on the cutoff level of the κ statistic commonly cited in the literature, a κ of 0.354 was interpreted as fair agreement [29]. Multimedia Appendix 5 shows the detailed interrater reliability results.

Table 2 shows the relationship between the MARS scores and general characteristics of the 7 apps included in the analysis. There was an association between the price of mHealth apps and the MARS scores of information sections, but this did not reach statistical significance (*P*=.08). The number of downloads was negatively correlated with the prices of apps, even though this did not reach statistical significance. Statistically significant associations among the 4 sections of MARS scale were observed, except for the information section.

**Table 2:** Correlation matrix among MARS scores, price, number of downloads, and average user rating.

Characteristics		MARS^a^ scores					Price		Number of downloads^b^		Mean user rating
		1	2	3	4						
**MARS scores**											
	Engagement	—^c^									
	Functionality	0.68^d^	—								
	Aesthetics	0.95^e^	0.80^f^	—							
	Information	0.68^g^	0.94^h^	0.82^i^	—						
Price		0.36	0.48	0.54	0.70^j^	—					
Number of downloads^b^		0.30	0.32	0.25	0.18	–0.40		—			
Average user rating		0.09	0.12	0.06	0.18	0.00		0.73^k^		—	

^a^MARS: Mobile App Rating Scale.

^b^Number of downloads was not available for the iOS platform.

^c^Not applicable.

^d^*P*=.09.

^e^*P*=.001.

^f^*P*=.03.

^g^*P*=.09.

^h^*P*=.002.

^i^*P*=.02.

^j^*P*=.08.

^k^*P*=.096.

## Discussion

This systematic review found there to be an acceptable quality of mHealth apps with a DDI check in Chinese app stores, with an average MARS score of 3.63. However, the quality of the information section was polarized among apps included in the review. Specifically, nearly half of the investigated apps that aimed to identify any significant interaction associated with concurrently administered drugs showed relatively poor quality in scientific information. On the other hand, the MCDEX mobile app, developed under the supervision of the China National Health Committee, demonstrated high quality in content accuracy and comprehensiveness, highlighting the importance of the scientific or clinical basis and accountability dimensions. To our best knowledge, this study was the first analysis to systematically evaluate apps for DDI checks in China and underscores the importance of regulation in the mHealth apps, which is becoming a major source of information for health care providers in China, based on our ongoing survey exploring physicians’ knowledge and sources of information on DDIs.

In this in-depth analysis, the information quality of mHealth apps with a DDI check feature showed great variety. The total MARS score ranged from 1.97 to 4.23, whereas the MARS score for the information section ranged from 1.41 to 4.60, suggesting there was a certain proportion of apps with relatively low quality. These findings were consistent with another app review conducted in Canadian app stores [9]. Furthermore, 4 out of 7 investigated apps failed to identify over 50% of the tested DDI pairs. The low quality of the information section can be partially explained by a lack of evidence. The MCDEX mobile app, which was based on the knowledge bases developed in China, demonstrated relatively high-quality scientific information. A moderate score in the information section was observed in 3 apps that used both product information (package inserts) and existing treatment guidelines as the major source of information, while a poor score was observed among those apps using product information as the sole source of DDI knowledge. The reason why this is a low-quality information source is that package inserts are very likely to be outdated and cannot provide the best-available evidence. In addition, clinicians or drug experts are not commonly involved in the process of app development. The DDI check feature could be helpful in preventing ADEs but can only operate properly when the medication list is accurate and complete. Therefore, these findings call for immediate action to address the low scientific quality of mHealth apps, which is a potential threat to patient safety.

Our study also suggested that prices could be an important factor influencing the information quality of mHealth apps with DDI features. The MCDEX mobile app, which required the highest subscription fee, scored highest in the information section by providing accurate and comprehensive information in DDIs. However, 4 apps available for free provided unsatisfactory drug information. Of further note, these free apps were very popular in the market, making patient safety a serious concern.

In this review, only 7 mHealth apps were identified in Chinese app stores, fewer than those available to Canadians in English (n=26) according to a similar review conducted in 2018 [9]. This may be explained by the fact that the Canadian review targeted consumer apps with DDI check features, but the DDI check function was not included in consumer apps in China. In comparison with apps found in Canadian app stores, the Chinese apps had slightly higher MARS scores (3.63 vs 3.05) [9]. A potential explanation for this is the fact that the Chinese apps target health care professionals and thus require more rigorous clinal evidence. In terms of information quality measured by MARS, considerable variation was also observed. One-third of the Canadian apps scored lower than 1 out of 5, but around 15% (1/7) of the apps in Chinese app stores had the same score. For the functionality and aesthetic sections, similar results were reported in the Chinese and Canadian apps, but the Chinese apps scored higher in the engagement section than did the Canadian apps. If the investigated apps are mainly used as a reference for DDI information, it is not necessary to have a high score in the engagement section because these apps do not intend to engage users to effect behavioral changes [14].

This study has the following strengths. First, this is the first systematic review for DDI-related apps using the advanced crawling method for ensuring a more comprehensive search [10]. This review focused on non-English apps rather than English apps that have already been assessed [10]. Second, by using tested DDI pairs with high clinical importance, we evaluated the accuracy and comprehensiveness of the DDI checks available in the investigated apps to ensure the assessment process was as objective as possible. Finally, this review was conducted by clinical pharmacists who have extensive experience in medicine, especially DDIs.

There are also several limitations to our study. First, we searched the apps at a certain time point, and we cannot exclude the possibility that newly released apps might have been missed in the search. Second, our search might not have been sufficiently thorough. However, 2 raters performed an independent review with the consultation of a third rater, and thus the possibility that certain apps were missed should have been minimal. Third, despite the efforts to make raters familiar with the MARS scale by watching videos and reading protocols, the rating scores might have been subjective, which makes it difficult to compare across different apps. A higher score reported for a certain app may not indicate higher quality; instead, it may suggest that this app was overscored by the raters. To address this concern, we also used 35 drug pairs to assess the information quality in a more objective way.

In conclusion, this study provided a comprehensive overview of the mHealth apps with a DDI check function available in Chinese app stores. Using the multidimensional framework for the evaluation of apps, we found that the quality of mHealth apps was acceptable although a limited number of apps provided inaccurate and incomplete information about DDIs. The majority of investigated apps provided accurate and comprehensive information. A few of the apps that had large number of downloads offered a relatively low quality of drug information. As most of the apps found in Chinese app stores targeted health care professionals who may use these apps as a reference for DDI information, our findings underscore the importance of providing accurate scientific information in mHealth apps, as DDIs can have serious consequences.

## Appendix

Multimedia Appendix 1 Mobile App Rating Scale (MARS).

Multimedia Appendix 2 List of drug–drug interaction pairs for the Mobile App Rating Scale (MARS) number 15 and 16.

Multimedia Appendix 3 Detailed results of information quality and accountability.

Multimedia Appendix 4 Detailed results of Mobile App Rating Scale (MARS) number 15 and 16.

Multimedia Appendix 5 Detailed interrater reliability results.

## Abbreviations

ADE: adverse drug event
DDI: drug–drug interaction
MARS: Mobile App Rating Scale
mHealth: mobile health
NMPA: National Medical Products Administration
PRISMA: Preferred Reporting Items for Systematic Reviews and Meta-Analyses
