# Supplementary material for: Mobile Apps for Drug–Drug Interaction Checks in Chinese App Stores: Systematic Review and Content Analysis
Source: JMIR Mhealth Uhealth. 2021 Jun 15;9(6):e26262. doi: 10.2196/26262 (PMC8277361; doi:10.2196/26262)
Supplement: Multimedia Appendix 2 [file mhealth_v9i6e26262_app2.docx]

**Appendix 2. List of drug-drug interaction pairs for the MARS #15 and #16**

| **Have interaction** |
| --- |
| Dexmethylphenidate Hydrochloride+Isocarboxazid |
| Atazanavir+Omeprazole |
| Fluoxetine+Tranylcypromine |
| Digoxin+Itraconazole |
| Irinotecan+Ritonavir |
| Irinotecan+Ketoconazole |
| Warfarin+Probenecid |
| TCA+Tranylcypromine |
| Amiodarone+Procainamide |
| Ramelteon+Fluvoxoxamine |
| Bosentan+Ritonavir |
| Simvastatin+Amiodarone |
| Simvastatin+Clarithromycin |
| Simvastatin+Itraconazole |
| Indinavir+Ergotamine |
| Clarithromycin+Ergotamine |
| Ketoconazole+Ergotamine |
| Tizanidine+Ciprofloxacin |
| Sumatriptansuccinate+Tranylcypromine |
| Warfarin+Sulfamethoxazole |
| Digoxin+Amiodarone |
| Sildenafil+Isosorbide mononitrate |
| Theophylline+Ciprofloxacin |
| Pimozide+Ketoconazole |
| Itraconazole+Quinidine |
| Warfarin+Fluconazole |
| Alprazolam+Itraconazole |
| Pethidine+Tranylcypromine |
| **No Interaction** |
| Codeine+Amoxicillin |
| Carbamazepine+Erythromycin |
| Metformin+Erythromycin |
| Digoxin+Sildenafil |
| Warfarin+Digoxin |
| Warfarin+Pravastatin |
| Fexofenadine hydrochloride+Metoprolol |
