# Supplementary material for: Mobile Apps for Drug–Drug Interaction Checks in Chinese App Stores: Systematic Review and Content Analysis
Source: JMIR Mhealth Uhealth. 2021 Jun 15;9(6):e26262. doi: 10.2196/26262 (PMC8277361; doi:10.2196/26262)
Supplement: Multimedia Appendix 3 [file mhealth_v9i6e26262_app3.docx]

**Appendix 3. Detailed results of Information quality and accountability**

| **App name** | **Concordance with existing evidence** | **Major source of information** | **Presence of citations** | **Clinician involvement** | **Affiliation with credible organization** | **Expert assessment** | **Regulatory approval** | **Copyright information** | **Developer** | **Date of last update** |
| --- | --- | --- | --- | --- | --- | --- | --- | --- | --- | --- |
| MCDEX mobile | Yes | Knowledge base, package insert, guidelines | Yes | Yes | Yes | Yes | No | Yes | Sichuan Medicom Software Co., Ltd. | 3/3/2020 |
| Medication Assistant plus by DXY | Yes | Package insert, guidelines, etc. | Yes | Yes | Yes | No | No | Yes | Guanlan Networks (Hangzhou) Co., Ltd. | 10/30/2020 |
| Medication Reference | No | Package insert, guidelines, etc. | No | No | Yes | No | No | Yes | Beijing Kingyee Technology Ltd | 5/19/2020 |
| Medication Assistant of People's Health | Yes | Package insert, guidelines, etc. | Yes | Yes | Yes | No | No | Yes | People's Medical Publishing House Co., Ltd | 7/20/2020 |
| Medication Guidelines | No | Package insert | No | No | No | No | No | No | Guangxi Yingteng Technology Co., Ltd. | 10/30/2020 |
| DXY | No | Package insert | No | Yes | Yes | Yes | No | Yes | Hangzhou Lianke Meixun Biology Pharmaceutical Technology Co., Ltd. | 11/15/2020 |
| Yi Mai Tong | No | Package insert | No | No | Yes | Yes | No | Yes | Beijing Kingyee Technology Co., Ltd | 11/6/2020 |
