# Supplementary material for: Mobile Apps for Drug–Drug Interaction Checks in Chinese App Stores: Systematic Review and Content Analysis
Source: JMIR Mhealth Uhealth. 2021 Jun 15;9(6):e26262. doi: 10.2196/26262 (PMC8277361; doi:10.2196/26262)
Supplement: Multimedia Appendix 4 [file mhealth_v9i6e26262_app4.docx]

**Appendix 4. Detailed results of MARS #15 and #16**

**Rater 1**

| **Name** | **A. Engagement** | | | | | | **B. Functionality** | | | | | **C. Aesthetics** | | | | **D. Information** | | | | | | | | **Mean** |
| --- | --- | --- | --- | --- | --- | --- | --- | --- | --- | --- | --- | --- | --- | --- | --- | --- | --- | --- | --- | --- | --- | --- | --- | --- |
|  | **1** | **2** | **3** | **4** | **5** | **Avg.** | **6** | **7** | **8** | **9** | **Avg.** | **10** | **11** | **12** | **Avg.** | **13** | **14** | **15** | **16** | **17** | **18** | **19** | **Avg.** |  |
| MCDEX mobile | 3 | 3 | 3 | 3 | 5 | 3.4 | 4 | 5 | 4 | 4 | 4.3 | 5 | 4 | 5 | 4.7 | 5 | 5 | 5 | 5 | N | 3 | N | 4.6 | **4.23** |
| Medication Reference | 1 | 2 | 2 | 4 | 4 | 2.6 | 4 | 5 | 4 | 4 | 4.3 | 3 | 3 | 4 | 3.3 | 5 | 5 | 3 | 3 | 4 | 3 | N | 3.9 | **3.51** |
| Medication Assistant by DXY | 2 | 3 | 4 | 4 | 4 | 3.4 | 4 | 4 | 4 | 4 | 4.0 | 5 | 5 | 3 | 4.3 | 5 | 5 | 3 | 4 | 4 | 3 | N | 4.0 | **3.94** |
| Yi Mai Tong | 4 | 3 | 2 | 2 | 4 | 3.0 | 4 | 4 | 4 | 4 | 4.0 | 4 | 4 | 3 | 3.7 | 5 | 5 | 2 | 3 | 3 | 3 | N | 3.5 | **3.54** |
| Medication Assistant of People's Health | 2 | 3 | 2 | 5 | 5 | 3.4 | 3 | 4 | 5 | 5 | 4.3 | 5 | 4 | 4 | 4.3 | 5 | 5 | 1 | 1 | 4 | 4 | N | 3.4 | **3.83** |
| DXY | 2 | 4 | 4 | 4 | 4 | 3.6 | 4 | 4 | 4 | 4 | 4.0 | 4 | 5 | 4 | 4.3 | 5 | 5 | 2 | 4 | 4 | 3 | N | 3.8 | **3.93** |
| Medication Guidelines | 1 | 2 | 1 | 2 | 3 | 3.0 | 4 | 5 | 1 | 4 | 3.5 | 4 | 3 | 3 | 3.3 | 4 | 5 | 0 | 0 | N | 3 | N | 2.4 | **3.93** |
| **Rater2** |  |  |  |  |  |  |  |  |  |  |  |  |  |  |  |  |  |  |  |  |  |  |  |  |
| **Name** | **A. Engagement** | | | | | | **B. Functionality** | | | | | **C. Aesthetics** | | | | **D. Information** | | | | | | | | **Mean** |
|  | **1** | **2** | **3** | **4** | **5** | **Avg.** | **6** | **7** | **8** | **9** | **Avg.** | **10** | **11** | **12** | **Avg.** | **13** | **14** | **15** | **16** | **17** | **18** | **19** | **Avg.** |  |
| MCDEX mobile | 3 | 3 | 3 | 4 | 4 | 3.4 | 5 | 5 | 5 | 4 | 4.8 | 5 | 4 | 4 | 4.3 | 5 | 4 | 5 | 5 | N | 4 | N | 4.6 | **4.27** |
| Medication Reference | 4 | 4 | 1 | 2 | 4 | 3.0 | 5 | 5 | 5 | 4 | 4.8 | 4 | 4 | 4 | 4.0 | 5 | 4 | 3 | 3 | N | 3 | N | 3.6 | **3.85** |
| Medication Assistant by DXY | 4 | 4 | 2 | 4 | 5 | 3.8 | 5 | 5 | 4 | 5 | 4.8 | 4 | 4 | 4 | 4.0 | 5 | 4 | 3 | 4 | N | 3 | N | 3.8 | **4.09** |
| Yi Mai Tong | 3 | 3 | 2 | 3 | 2 | 2.6 | 4 | 2 | 4 | 4 | 3.5 | 3 | 3 | 3 | 3.0 | 2 | 3 | 2 | 3 | N | 3 | N | 2.6 | **2.92** |
| Medication Assistant of People's Health | 4 | 3 | 2 | 4 | 4 | 3.4 | 4 | 4 | 4 | 4 | 4.0 | 4 | 4 | 4 | 4.0 | 5 | 4 | 1 | 1 | 4 | 3 | N | 3.0 | **3.60** |
| DXY | 5 | 4 | 4 | 4 | 3 | 4.0 | 5 | 3 | 4 | 5 | 4.3 | 5 | 5 | 4 | 4.7 | 4 | 4 | 2 | 4 | N | 3 | N | 3.3 | **4.06** |
| Medication Guidelines | 2 | 2 | 1 | 1 | 2 | 1.6 | 3 | 4 | 1 | 2 | 2.5 | 3 | 2 | 2 | 2.3 | 2 | 3 | 0 | 0 | N | 2 | N | 1.4 | **1.96** |
