# Supplementary material for: Mobile Apps for Drug–Drug Interaction Checks in Chinese App Stores: Systematic Review and Content Analysis
Source: JMIR Mhealth Uhealth. 2021 Jun 15;9(6):e26262. doi: 10.2196/26262 (PMC8277361; doi:10.2196/26262)
Supplement: Multimedia Appendix 5 [file mhealth_v9i6e26262_app5.docx]

**Appendix 5. Detailed inter-rater reliability results**

| **Method** | **Co eff** | **Std Err** | **95% C.I.** | **P-Value** |
| --- | --- | --- | --- | --- |
| Cohen’s Kappa | 0.354 | 0.06 | (0.236 - 0.473) | <0.001 |
| Fleiss' Kappa | 0.353 | 0.06 | (0.234 - 0.472) | <0.001 |
| Krippendorff's Alpha | 0.356 | 0.06 | (0.237 - 0.475) | <0.001 |
| Brennan-Prediger | 0.449 | 0.047 | (0.357 - 0.542) | <0.001 |
| Percent Agreement | 0.489 | 0.044 | (0.403 - 0.575) | <0.001 |
| AC1 | 0.456 | 0.046 | (0.364 - 0.547) | <0.001 |
